# Soil metagenome datasets underneath the Arecibo Observatory reflector dish

**DOI:** 10.1016/j.dib.2019.104710

**Published:** 2019-10-24

**Authors:** Miguel G. Rodriguez-Reyes, Carlos Rios-Velazquez

**Affiliations:** University of Puerto Rico at Mayagüez, Puerto Rico

**Keywords:** Soil metagenomics, Metagenome, Arecibo observatory, Reflector dish

## Abstract

The Arecibo Observatory (AO) located in Arecibo, Puerto Rico, is the most sensitive, powerful and active planetary radar system in the world [1]. One of its principal components is the 305 m-diameter spherical reflector dish (AORD), which is exposed to high frequency electromagnetic waves. To unravel the microbial communities that inhabit this environment, soil samples from underneath the AORD were collected, DNA extracted, and sequenced using Illumina MiSeq. Taxonomic and functional profiles were generated using the MG-RAST server. The most abundant domain was Bacteria (91%), followed by Virus (8%), Archaea (0.9%) and Eukaryota (0.9%). The most abundant phylum was Proteobacteria (54%), followed by Actinobacteria (8%), Bacteroidetes (5%) and Firmicutes (4%). In terms of functions, the most abundant among the metagenome corresponded to phages, transposable elements and plasmids (16%), followed by clustering-based subsystems (11%), carbohydrates (10%), and amino acids and derivatives (9%). This is the first soil metagenomic dataset from dish antennas and radar systems, specifically, underneath the AORD. Data can be used to explore the effect of high frequency electromagnetic waves in soil microbial composition, as well as the possibility of finding bioprospects with potential biomedical and biotechnological applications.

**Table:** Specifications Table

Subject area	*Biology*
More specific subject area	*Metagenomics*
Type of data	*FASTQ files, figures*
How data was acquired	*Illumina MiSeq, MG-RAST*
Data format	*Raw, annotated*
Experimental factors	*Environmental sample*
Experimental features	*Metagenomic DNA was extracted from soil samples, shotgun-sequenced using Illumina and processed using MG-RAST*
Data source location	*Soil underneath the Arecibo Observatory 305 m Reflector Dish, Arecibo, Puerto Rico, (18.3454, -66.7526; 18.3448, -66.7518; 18.3436, -66.7521; 18.3439, -66.7534; 18.3442, -66.7529; 18.3441, -66.7527; 18.3442, -66.7524; 18.3443, -66.7527)*
Data accessibility	*Data of this metagenome is deposited in the NCBI database under BioProject PRJNA564120 (*https://www.ncbi.nlm.nih.gov/bioproject/PRJNA564120*); annotations are available in the MG-RAST server under Study ID mgp85324 (*https://www.mg-rast.org/linkin.cgi?project=mgp85324*)*.

**Table:** 

**Value of the Data** • To our knowledge, this is the first soil metagenomic dataset from dish antennas and radar systems, specifically, underneath the AORD. • This data can be compared to other soil types and antennas sites to explore how electromagnetic waves might impact the microbial communities. • This project have bioprospecting value to find microorganisms and genes of potential biomedical and biotechnological applications.

## 1 Data

**Fig. 1:**
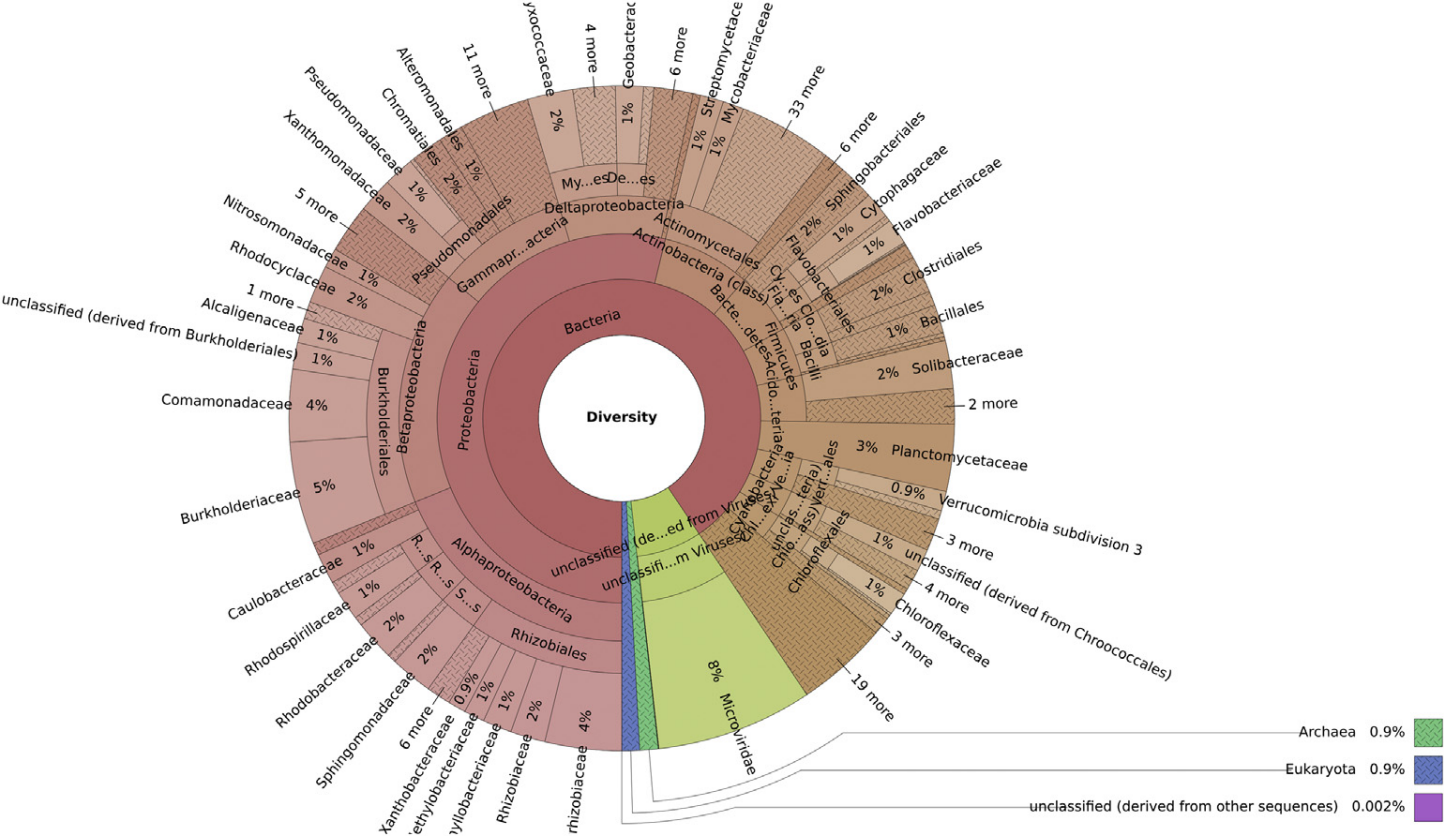
Taxonomic diversity of the AORD soil metagenome. The metagenome shows Bacteria as the most abundant domain (91%), followed by Virus (8%), Archaea (0.9%), Eukaryota (0.9%) and other sequences (0.002%). From the 38 bacterial phyla detected, the most abundant was Proteobacteria (54%), followed by Actinobacteria (8%), Bacteroidetes (5%), Firmicutes (4%), Acidobacteria (4%), and other 33 phyla that represents the remaining 25%. Among the bacteria, 100 orders were detected, from which the most abundant were Burkholderiales (12%), followed by Rhizobiales (10%), Actinomycetales (7%), Myxococcales (4%), and other 96 orders that represents the remaining 67%.

**Fig. 2:**
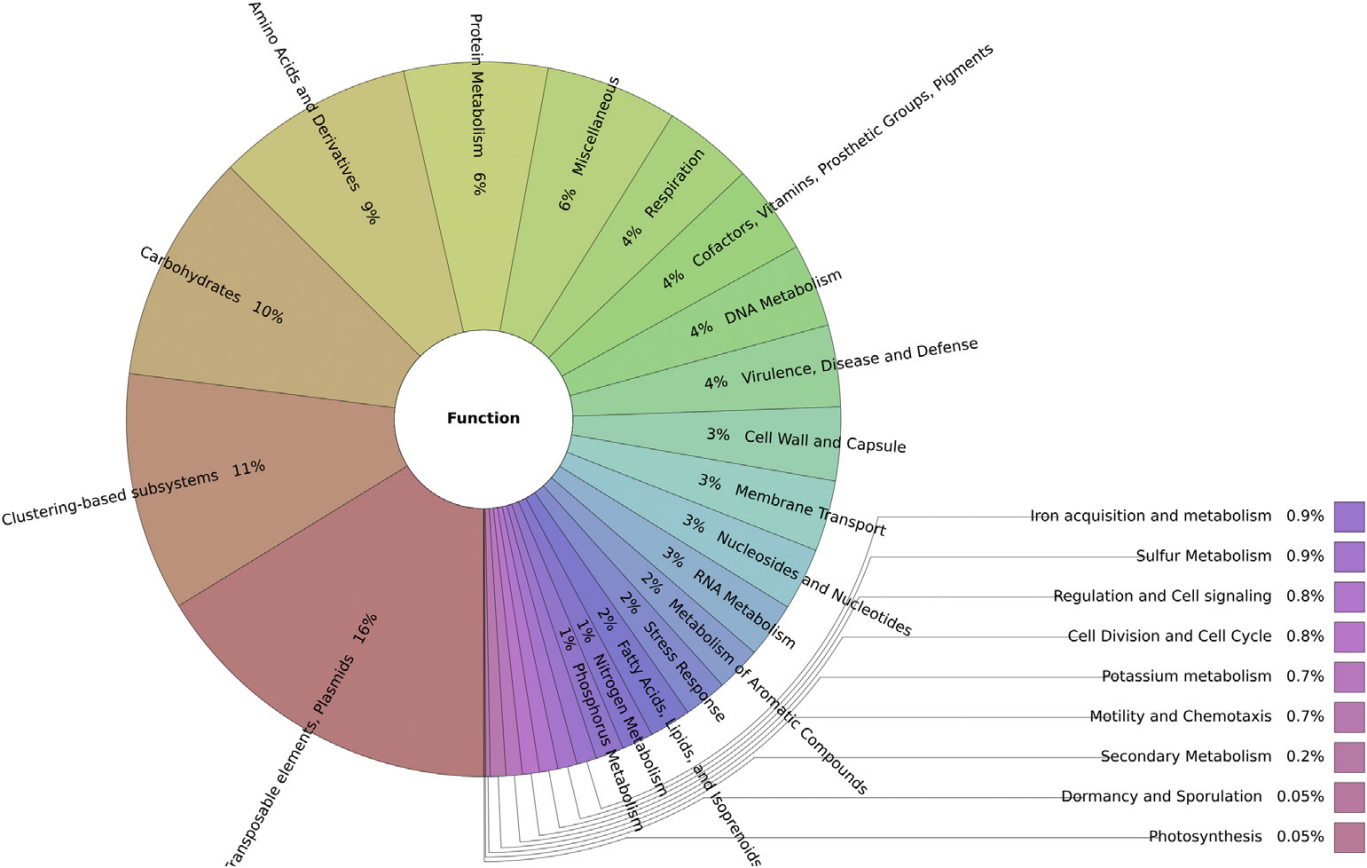
Functional profile of the AORD soil metagenome. The most abundant function among the AORD metagenome corresponded to phages, transposable elements and plasmids (16%), followed by clustering-based subsystems (11%), carbohydrates (10%), amino acids and derivatives (9%), protein metabolism (6%), miscellaneous (6%), respiration (4%), DNA metabolism (4%), virulence, disease and defense (4%) among other categories which represents the remaining 30%.

**Fig. 3:**
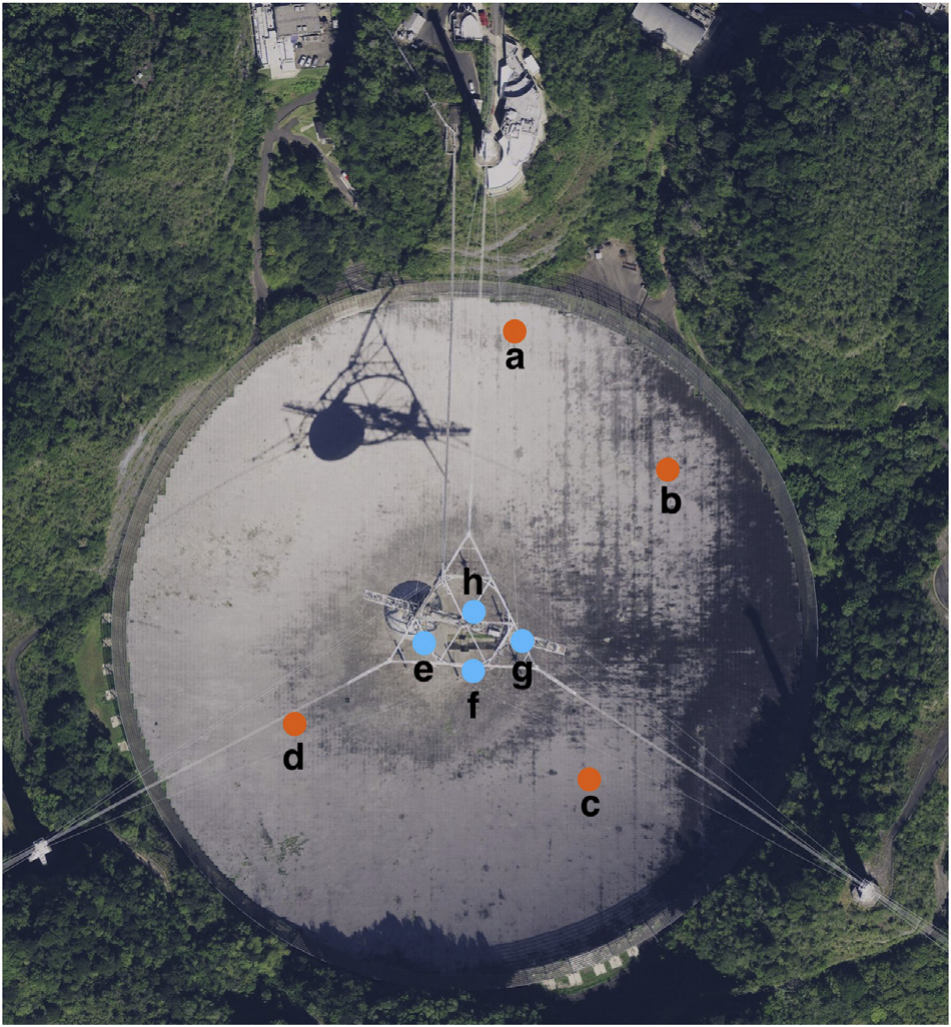
Soil sampling sites underneath the AORD. Peripheral soil samples (orange) and central soil samples (blue) (*Source*: Maps, Apple).

The Arecibo Observatory (AO), located in Arecibo, Puerto Rico (18.3442, −66.7526), is the most sensitive, powerful and active planetary radar system in the world [1]. One of the principal components of the AO is its 305 m-diameter spherical reflector dish (AORD). It is made of ∼40,000 perforated aluminum panels supported by steel cables over a natural karst sinkhole in a lowland moist and wet seasonal evergreen and semi-deciduous forest [2,3]. This reflective surface allows radio emissions originating from the sky to be focused into the antennas, and redirects radar waves to objects in the solar system. The AO operates at frequencies from 50 to 10,000 MHz, which have been shown to have adverse effects on microbial growth [[4], [5], [6]]. Even though these electromagnetic waves might not reach the microorganisms in the soil underneath the AORD, the effect of the influx (due to rain) of the ones that are exposed on top is unknown. For this study, we sampled the soil underneath the AORD (Fig. 3), sequenced the metagenome, and described the diversity (Fig. 1) and functional (Fig. 2) profiles of the microbial communities. This dataset containing raw FASTQ files and figures, is part of the first study that assesses the microbial community underneath the AORD.

## 2 Experimental design, materials, and methods

### 2.1 Sampling

Eight soil samples were collected (20 g each at 5 cm depth) from underneath the AORD: four from the periphery (a) 18.3454, −66.7526; (b) 18.3448, −66.7518; (c) 18.3436, −66.7521; (d) 18.3439, −66.7534 and four from the center (e) 18.3442, −66.7529; (f) 18.3441, −66.7527; (g) 18.3442, −66.7524; (h) 18.3443, −66.7527 (Fig. 3). Temperature of the sampling site ranged from 25.5 °C to 29.8 °C, and pH ranged from 7.32 to 8.49.

### 2.2 DNA extraction

Metagenomic DNA of the eight soil samples was extracted individually using the PowerSoil® DNA Isolation Kit (MO BIO Laboratories) following the manufacturer's protocol, except that for each sample, 0.30 g of soil was used and DNA was resuspended in 50 μL of TE1X (Tris-EDTA: 10 mM Tris-HCl, 1 mM EDTA; pH 8.0).

### 2.3 Metagenome sequencing

The extracted DNA was pooled and then sequenced at the Molecular Research DNA Laboratory (MR DNA, Shallowater, TX, USA, www.mrdnalab.com). The initial concentration of the pooled sample was 13.20 ng/μL measured using the Qubit™ dsDNA HS Assay Kit (Life Technologies). A genomic library was constructed with 50 ng of the pooled sample using the Nextera DNA Sample Preparation Kit (Illumina). After fragmentation and addition of adapter sequences, the final concentration of the library was 14.60 ng/μL using the Qubit® dsDNA HS Assay Kit (Life Technologies) with an average length of 1273 bp using the Agilent 2100 Bioanalyzer (Agilent Technologies). The library was diluted to 12.0 pM and pair-end-sequenced using the Illumina MiSeq Reagent Kit v3 for 600 cycles. Sequences were preprocessed using FastQC [7] as quality control check, and the FASTX toolkit [8] to remove adapter sequences (FASTQ Clipper) and trim portions of sequences with Phred score < 30 (FASTQ Quality Trimmer).

### 2.4 Taxonomic and functional profiling

Processed sequences were uploaded to the Metagenomics Rapid Annotation using Subsystems Technology server (MG-RAST, www.mg-rast.org) [9]. The *in silico* profile generated for the microbial community underneath the AORD using MG-RAST, includes classification based on its taxonomic diversity (RefSeq) and genes’ functionality (Subsystems, level 1) (Fig. 1, Fig. 2).

## References

[1] Zambrano Marin L.F., Virkki A.K., Marshall S., Venditti F., Taylor P.A., Rivera Valentin E.G., Aponte B. (2019). Arecibo observatory radar observations: 2017-2018. Am. Astron. Soc. Meet. Abstr..

[2] Telescope description. https://www.naic.edu/ao/telescope-description.

[3] Helmer E., Ramos O., López T.D.M., Quinones M., Diaz W. (2002). Mapping the forest type and land cover of Puerto Rico, a component of the Caribbean biodiversity hotspot Caribb. J. Sci..

[4] Coskun O., Sofu A., Kahriman M., Comlekci S. (2016). The effects of electromagnetic fields with a frequency of 2450 MHz on E. coli bacteria. J. of Biomim., Biomater. and Biomed. Eng..

[5] Salmen S.H., Alharbi S.A., Faden A.A., Wainwright M. (2018). Evaluation of effect of high frequency electromagnetic field on growth and antibiotic sensitivity of bacteria Saudi. J. Biol. Sci. (Bombay).

[6] Tadevosian A., Kalantarian V., Trchunian A. (2007). The effects of electromagnetic radiation of extremely high frequency and low intensity on the growth rate of bacteria Escherichia coli and the role of medium pH. Biofiz..

[7] FastQC http://www.bioinformatics.babraham.ac.uk/projects/fastqc/.

[8] FASTX http://hannonlab.cshl.edu/fastx_toolkit/.

[9] Meyer F., Paarmann D., D'Souza M., Olson R., Glass E.M., Kubal M., Wilkening J. (2008). The metagenomics RAST server –a public resource for the automatic phylogenetic and functional analysis of metagenomes. BMC Bioinf..

